# Sustained Biotic-Abiotic Hybrids Methanogenesis Enabled Using Metal-Free Black Phosphorus/Carbon Nitride

**DOI:** 10.3389/fmicb.2022.957066

**Published:** 2022-07-12

**Authors:** Andong Hu, Tao Fu, Guoping Ren, Minghan Zhuang, Weiqi Yuan, Sining Zhong, Shungui Zhou

**Affiliations:** Fujian Provincial Key Laboratory of Soil Environmental Health and Regulation, College of Resources and Environment, Fujian Agriculture and Forestry University, Fuzhou, China

**Keywords:** biotic-abiotic hybrid systems, metal-free photocatalysts, multicycle methanogenesis, methanation of CO_2_, photogenerated electron-hole separation

## Abstract

Biotic-abiotic hybrid systems (BAHs) constructed by integrating biological methanogens with photocatalysts offer novel approaches for the effective solar-driven conversion of CO_2_ to CH_4_, providing significant inspiration for achieving carbon neutrality and alleviating the energy crisis. As metal photocatalysts would cause photocorrosion that damages microbial cells and lead to system imbalance. Therefore, exploring suitable metal-free photocatalysts is of particular importance in the search for more efficient and sustainable BAHs to improve the actual operability and applicability. Herein, black phosphorus/carbon nitride (BPCN*_*x*_*) as an alternative metal-free heterostructure was combined with *Methanosarcina barkeri* (*M. barkeri*) to construct *M. barkeri*-BPCN*_*x*_* hybrid systems, and their cyclic methanogenesis performance was investigated. Our results demonstrated that BPCN*_*x*_* promotes the separation of photogenerated charges and enhances the quantum yield, providing a sustained energy source for the cyclically driven *M. barkeri* reduction of CO_2_ to CH_4_ under visible light. Our system achieved a total CH_4_ yield of 1087.45 ± 29.14 μmol g_cat_^–1^ after three cycles, 1.96 times higher than that of *M. barkeri*-Ni@CdS. *M. barkeri*-BPCN*_*x*_* overcame the defects of the metal photocatalyst and kept cell permeability, achieving cyclic stability and effectively maintaining the activity of *M. barkeri*. These results highlight the viable role of BPCN*_*x*_* as a metal-free photocatalysts in the construction of BAHs for the sustained and efficient methanation of CO_2_, which is conducive to the development of an environmentally-friendly, low-cost, and efficient strategy for the conversion of CO_2_ to CH_4_.

## Introduction

The excessive consumption of fossil fuels will not only lead to a shortage of non-renewable resources but also release large amounts of greenhouse gases such as carbon dioxide (CO_2_) into the atmosphere, causing a series of environmental problems (Kong et al., 2020). Energy and climate solutions aiming at carbon neutrality are new requirements for future sustainable development, and the conversion of CO_2_ into high-value energy substances is an effective way to achieve this goal, alleviating or even solving environmental pollution and energy crises (Gil and Bernardo, 2020; Zhang et al., 2022). As a clean and efficient carbon-based fuel, methane (CH_4_) is considered an effective tool to realize CO_2_ resource utilization, providing a strategy for the development of new energy (Shi et al., 2022).

Thus far, researchers have focused on developing new technologies and catalysts to achieve the efficient photocatalytic conversion of CO_2_ to CH_4_. Abiotic photocatalytic systems, which use light energy to reduce CO_2_ to produce high value-added substances, have attracted extensive attention due to their simple structure and high designability (Tong et al., 2012). However, the low utilization efficiency of solar energy results in the poor performance and product selectivity of photocatalytic systems. To address this technical bottleneck, many researchers have proposed biotic-abiotic hybrid systems (BAHs) that utilize microorganisms and photocatalysts with self-healing and self-replication characteristics, demonstrating less energy loss and higher product selectivity under complex environmental conditions (Sakimoto et al., 2016; Cestellos-Blanco et al., 2020). Notably, recent studies have reported the successful construction of BAHs using cadmium sulfide (CdS) metal photocatalysts (Ye et al., 2019). As the methanogenesis efficiency was greatly limited by the rapid reorganization of CdS photogenerated charges, nickel-doped cadmium sulfide (Ni@CdS) was used to improve the electron–hole separation efficiency to enhance their methanogenesis performance (Ye et al., 2020). Although the introduction of Ni can overcome the issue caused by CdS photogenerated electron separation, the metal photocatalyst itself is prone to photocorrosion, heavy metal release, and microbial poisoning, which are still key problems that result in the instability and non-cyclability of BAHs (Ye et al., 2021).

Biofriendly metal-free photocatalysts are expected to replace metal photocatalysts to address the unsustainability of BAHs (Athira et al., 2021). As a potential representative, carbon nitride (CN*_*x*_*) has attracted widespread attention with its advantages of medium bandgap, non-toxicity, and powerful photocatalytic potential (Mishra et al., 2019; Hao et al., 2020; Adekoya et al., 2021). However, CN*_*x*_* usually needs to be loaded with noble metals to exhibit its excellent photocatalytic performance, due to the limited efficiency of photogenerated electron–hole separation (Ma et al., 2016; Alaghmandfard and Ghandi, 2022). To overcome this deficiency, metal-free black phosphorus (BP) can efficiently realize the function of metal cocatalysts (e.g., Ni and Cu) due to its direct band gap and good light absorption efficiency (Shen et al., 2020). Therefore, combining BP with CN*_*x*_* may lower the potential barrier, improve the separation and migration of photogenerated electrons, and inhibit the recombination of electron–hole pairs, thereby enhancing the photocatalytic performance and ideal product selectivity of the photocatalysts for use in BAHs (Zheng et al., 2020). In addition, the integration of metal-free black phosphorus/carbon nitride (BPCN*_*x*_*) with microorganisms will likely overcome the defects of metal photocatalysts and allow for stable and sustainable systems. To this end, we hope to construct metal-free BAHs and explore whether BPCN*_*x*_* can achieve excellent CO_2_ methanation performance.

Herein, BPCN*_*x*_* was selected as a potential metal-free photocatalyst and combined with *Methanosarcina barkeri* for the construction of *M. barkeri*-BPCN*_*x*_* hybrid systems with cyclic methanogenesis performance. The fast charge separation performance of BPCN*_*x*_* was verified, and the photoelectric and methanogenesis properties of *M. barkeri*-BPCN*_*x*_* after the introduction of BPCN*_*x*_* were systematically evaluated. In addition, the cyclic methanogenesis performance and system stability of *M. barkeri*-BPCN*_*x*_* were evaluated by comparing BAHs constructed from metals, thereby revealing the potential mechanism for realizing the cyclically driven reduction of CO_2_ to CH_4_. This study will provide important implications for the development of environmentally-friendly, low-cost, and effectively stable BAHs.

## Materials and Methods

### Synthesis of CN*_*x*_* Photocatalysts

Amine-functionalized polymeric carbon nitride (^H2N^CN*_*x*_*) was first polymerized in a muffle furnace at 550°C for 4 h using melamine. Cyanamide functionalized polymeric carbon nitride (^NCN^CN*_*x*_*) was then synthesized by grinding ^H2N^CN*_*x*_* and potassium thiocyanate (KSCN) followed by calcination at 400°C for 1 h and again at 500°C for 30 min in an Ar atmosphere tube furnace (Kasap et al., 2018). Finally, ^NCN^CN*_*x*_* was ground and washed multiple times with oxygen-free water to remove residual KSCN and dried under vacuum at 60°C. The following experiments all used ^NCN^CN*_*x*_* (CN*_*x*_*).

### Preparation of BP Nanosheets

Black phosphorus powder (99.998%) was purchased from Zhongke Materials (Wuhan Institute of Advanced Technology, Chinese Academy of Sciences, Beijing, China). First, BP (500 mg) was added to 50 mL of N-methylpyrrolidone (NMP), ultrasonicated in a water bath for 8 h (temperature-controlled below 25°C), and centrifuged (1,000 rpm for 3 min) to remove larger BP particles. The obtained supernatant was then washed by centrifugation (14,000 rpm for 10 min) to remove NMP, and the washed powder was vacuum freeze-dried for 24 h to obtain two-dimensional BP nanosheets. As shown in Supplementary Figure 1, comparing the XRD patterns of BP powder and nanosheets before and after preparation showed that the prepared black phosphorus nanosheets were structurally stable.

### Preparation of BPCN*_*x*_* Photocatalysts

The prepared BP nanosheets and CN*_*x*_* were added to an anaerobic bottle containing oxygen-free water in a certain proportion. After sonication for 2 h (temperature-controlled below 25°C), the BPCN*_*x*_* mixture was subsequently stirred for 1 h. Finally, the samples were vacuum freeze-dried overnight to obtain BPCN*_*x*_*. All sampling operations were carried out in an anaerobic glove box (Bugbox, Ruskinn Technology Ltd., United Kingdom) to ensure anaerobic conditions.

### Construction of *Methanosarcina barkeri*-BPCN*_*x*_*

*Methanosarcina barkeri* MS (DSM 800) was purchased from DSMZ (Braunschweig, Germany). The obtained *M. barkeri* was added to sterilized heterotrophic medium (Supplementary Table 1) with acetic acid as a carbon source and placed in a constant temperature incubator at 35 ± 2°C for logarithmic phase growth (OD_600_ ≈ 0.2) (Ye et al., 2019). The prepared BPCN*_*x*_* was then added to construct the *M. barkeri*-BPCN*_*x*_* hybrid systems. After incubation in the dark for 2 days, the suspension was centrifuged at 7,500 rpm at 4°C for 6 min to remove the supernatant and washed three times with 0.9% NaCl to remove residual NaAc and Na_2_S⋅9H_2_O. The final precipitation was resuspended in 0.9% NaCl solution (5 mL) and 50 mL of sterilized autotrophic medium (SAM) was added (Supplementary Table 1), using NaHCO_3_ as a carbon source and 0.15 wt% cysteine (Cys) as a sacrificial reagent to quench holes (Wang et al., 2017). In multi-cycle CH_4_ production experiments, the medium was refreshed and supplemented with an equal amount of Cys every 5 days. To ensure strict anaerobic conditions, all cultivation and sampling operations were performed in an anaerobic glovebox with a gas mixture of 80% N_2_ and 20% CO_2_ (vol/vol).

The performance of *M. barkeri*-BPCN*_*x*_* in the reduction of CO_2_ to CH_4_ was studied under simulated LED illumination (395 ± 5 nm; 0.8 ± 0.2 mW cm^–2^). A controlled experiment was set up to evaluate the roles of *M. barkeri*, BPCN*_*x*_*, and light in CH_4_ production. The CH_4_ production performance of *M. barkeri*-BPCN*_*x*_* under different weight ratios of BP to CN*_*x*_* (1, 3, 6, and 10 wt%) and light:dark cycles (12 h:12 h) was investigated. Among them, the 6 wt% weight ratio of BP to CN*_*x*_* had the highest CH_4_ yield, which is expressed as BPCN*_*x*_* herein below (Supplementary Figure 2). In addition, the stability of *M. barkeri*-BPCN*_*x*_* for methanogenesis was investigated with three successive 5-day cycles (i.e., a total of 15 days) by refreshing the medium *in situ* and compared with *M. barkeri*-Ni@CdS. The concentration of CH_4_ was measured using a Shimadzu GC2014 gas chromatograph equipped with a Porapak Q column (2 m × 3 mm) and a flame ionization detector (FID). Nitrogen and hydrogen were used as the carrier and combustion gas, respectively, and the injection volume was 100 μL. The quantum yield (QY) of *M. barkeri*-BPCN*_*x*_* was calculated as previously reported (Chen et al., 2022). In addition, to verify the source of CH_4_ production, a control experiment was set up to replace NaH^12^CO_3_ in the medium with NaH^13^CO_3_. Then, headspace gas mass spectra were determined using an Agilent 7890-5975c gas chromatograph–mass spectrometer in the selected ion monitoring (SIM) mode (m/z = 31, 46).

### Characterization

The *M. barkeri*-BPCN*_*x*_* samples were fixed (12 h) with 2.5% pentanediol, eluted with ethanol gradients (30, 50, 70, 80, 90, and 95%), and finally stored in 100% ethanol (Wang et al., 2019). The morphology and structure of samples were measured with a Hitachi SU8020 scanning electron microscope and a Tecnai G2 F20 S-TWIN transmission electron microscope. The X-ray diffraction patterns were detected using a Shimadzu XRD-6000 and recorded in the 2θ range of 5–80° at a scan speed of 1° min^–1^. The energy and valence band (VB) spectra were measured using an American Thermo ESCALA 250 X-ray photoelectron spectrometer system with Al Kα radiation at 30 eV and fitted by X-ray photoelectron spectroscopy (XPS) PEAK41 software.

The diffuse reflectance spectra of *M. barkeri*-BPCN*_*x*_* were measured using a Shimadzu UV2600 UV-Vis spectrometer. Photocurrent (*I*-*t*) and electrochemical impedance spectroscopy (EIS) measurements were taken on a Shanghai Chenhua CHI 660E electrochemical workstation, where an ITO conductive glass slide (1 cm × 4 cm) was the working electrode and platinum and saturated calomel electrodes were respectively the counter and reference electrodes. Microbial live/dead staining was performed using a Live/Dead BacLight™ kit, and images were acquired using a Zeiss LSM880 confocal laser scanning microscope. The redox capacity of *M. barkeri* in BAHs was evaluated by 2,3,5-triphenyl tetrazolium chloride (TTC) staining, as described in previous research reports (Chen et al., 2020). By setting up ONPG hydrolysis experiments, the absorbance at 405 nm was measured by UV-Vis spectroscopy to characterize the permeability of the intracellular membrane (Yong et al., 2013). An NPN absorption experiment was established to characterize the permeability of the outer cell membrane by fluorescence spectroscopy (emission wavelength 370–500 nm, excitation wavelength 355 nm) (Liu et al., 2012).

All experiments were performed in triplicate. Differences were evaluated using the Student’s *t*-test, where a *p*-value < 0.05 was considered statistically significant.

## Results and Discussion

### Synthesis of the BPCN*_*x*_* Metal-Free Photocatalyst

**FIGURE 1:**
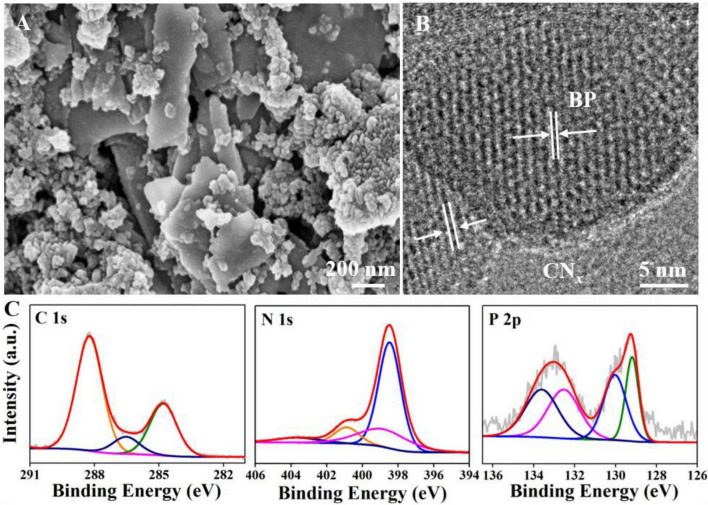
Characterizations of BPCN*_*x*_*. **(A)** SEM image. **(B)** TEM image. **(C)** XPS C 1s, N 1s, and P 2p spectra.

The scanning electron microscopy (SEM) results showed that BP had a typical sheet-like structure and that CN*_*x*_* exhibited granular aggregates (Supplementary Figures 3A,B). The specific structures of CN*_*x*_* and BP were simultaneously observed in the BPCN*_*x*_* images, indicating that the materials had successfully formed a composite (Figure 1A). To further confirm the formation of BPCN*_*x*_*, we characterized BPCN*_*x*_* by high-resolution transmission electron microscopy (TEM). BP displayed clear lattice fringes, with the lattice spacings of 0.256 and 0.333 nm respectively corresponding to the 040 and 021 crystal planes of BP (Supplementary Figure 3C), whereas CN*_*x*_* had no lattice fringes in the amorphous state (Zhu et al., 2017). The BPCN*_*x*_* image revealed that the BP lattice fringes were surrounded by amorphous CN*_*x*_* regions, indicating that BP had established intimate contact at the CN*_*x*_* junctions (Figure 1B). Furthermore, the channel formed by the tight attachment between BP and CN*_*x*_* had a positive effect on charge transfer (He et al., 2020). On this basis, high-resolution XPS was used to obtain the electron energy spectra and chemical information of BPCN*_*x*_* (Figure 1C). Among them, a new peak was observed at about 132.5 eV in BPCN*_*x*_*, which can be attributed to the P–N bond of P_3_N_5_ (Zhu et al., 2017). Compared to reported results, the C 1s and P 2p peaks of BPCN*_*x*_*, respectively, shifted to higher and lower binding energies by about 0.1 and 0.65 eV, due to electron transfer between the photocatalysts (He et al., 2017; Hao et al., 2018). In theory, two photocatalysts with different Fermi energy levels (EFs) combine to form a heterojunction, and electrons would then transfer from higher to lower EFs until the system reaches equilibrium (Yang, 2021). Therefore, electrons could be transferred from CN*_*x*_* to BP in BPCN*_*x*_* through the above process.

### Construction of *M. barkeri*-BPCN*_*x*_* Hybrid Systems

**FIGURE 2:**
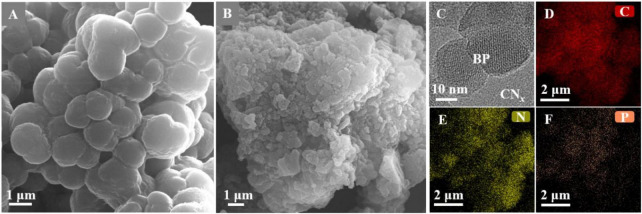
Combination of *M. barkeri* and BPCN*_*x*_*. **(A,B)** SEM images of pure *M. barkeri* and *M. barkeri*-BPCN*_*x*_*. **(C)** TEM images of *M. barkeri*-BPCN*_*x*_*. **(D–F)** EDS mapping of *M. barkeri*-BPCN*_*x*_*.

The combination of *M. barkeri* and BPCN*_*x*_* was verified through a variety of characterization methods. Compared with *M. barkeri*, the addition of BPCN*_*x*_* photocatalysts showed a rougher surface, indicating that the bacterial surface was successfully attached to the materials (Figures 2A,B). As shown in the TEM images (Figure 2C), the specific material properties of CN*_*x*_* and BP confirmed that BPCN*_*x*_* had combined with *M. barkeri*. The elemental composition of the surface-attached materials was confirmed by energy-dispersive X-ray spectroscopy (EDS mapping), and the results showed that the surface materials were mainly composed of carbon (C), nitrogen (N), and phosphorus (P) (Figures 2D–F). These results were consistent with the constituent elements of BPCN*_*x*_* as well as the XPS and XRD characterizations (Supplementary Figures 4A,B). The above data revealed that the successful construction of the *M. barkeri*-BPCN*_*x*_* had provided the foundation for the realization of CO_2_-to-CH_4_ conversion.

### Introduction of BPCN*_*x*_* to Enhance the Photoelectronic Properties of *M. barkeri*-BPCN*_*x*_*

**FIGURE 3:**
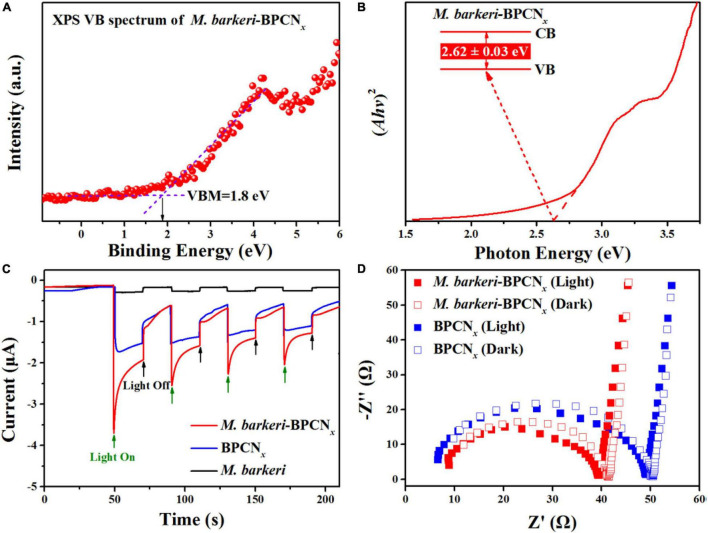
Photoelectrochemical tests of *M. barkeri*-BPCN*_*x*_*. **(A)** VB spectrum of *M. barkeri*-BPCN*_*x*_*. **(B)** Band gap plot of *M. barkeri*-BPCN*_*x*_*. **(C)** *I-t* curves with a light on/off cycle (20/20 s). **(D)** EIS Nyquist plots.

The photoelectronic properties of *M. barkeri*-BPCN*_*x*_* are essential to their function. A variety of electrochemical analyses were used to characterize the optical and electrical properties of the *M. barkeri*-BPCN*_*x*_* studied. The band structures of *M. barkeri*-BPCN*_*x*_* were obtained from the XPS valence band and UV-Vis solid diffuse reflectance spectra (Figures 3A,B). Moreover, BP and CN*_*x*_* could form a typical type I heterojunction (Low et al., 2017), with the e^–^ of the CN*_*x*_* conduction band transferring to the conduction band of BP, and the h^+^ of the CN*_*x*_* valence band transferring to the valence band of BP under visible light irradiation. This was conducive to the efficient separation and transport of light-induced e^–^–h^+^ pairs. Therefore, compared with the reported *M. barkeri*-CdS (2.69 eV) (Ye et al., 2019), *M. barkeri*-BPCN*_*x*_* displayed a lower bandgap energy (*E*_*g*_) of about 2.62 ± 0.03 eV, indicating that lower light energy input can be achieved through electronic transitions that help maintain the stability of *M. barkeri*-BPCN*_*x*_*. The estimated energy bands with the lowest unoccupied molecular orbital (LUMO) of -0.82 eV (vs. NHE) met the redox potential required for the reduction of CO_2_ to CH_4_ (Sun et al., 2018). To more intuitively characterize the photoelectronic properties of the reaction system, the *I*-*t* curve was used to characterize the current generated by *M. barkeri*-BPCN*_*x*_* under illumination, and the photocurrent was measured by alternating light:dark cycles. As shown in Figure 3C, the photocurrent of the reaction systems increased immediately to about 3.6 μA after turning on the light and quickly returned to its initial state after turning off the light. Compared with BPCN*_*x*_*, *M. barkeri*-BPCN*_*x*_* showed a stronger photocurrent response. The photoexcited e^–^–h^+^ pair exhibited a significantly prolonged lifetime after the addition of *M. barkeri* due to the higher separation efficiency (Ye et al., 2020). The electrical conductivity of the system was characterized by EIS. Compared with the dark reaction, the impedance of *M. barkeri*-BPCN*_*x*_* decreased under light irradiation, indicating the strong electrical conductivity of the reaction system (Figure 3D). The constructed *M. barkeri*-BPCN*_*x*_* required a lower photoexcitation energy and had excellent photogenerated electron separation ability, providing favorable conditions for the cyclically driven reduction of CO_2_ to CH_4_.

### Methanogenesis Performance of *M. barkeri*-BPCN*_*x*_*

**FIGURE 4:**
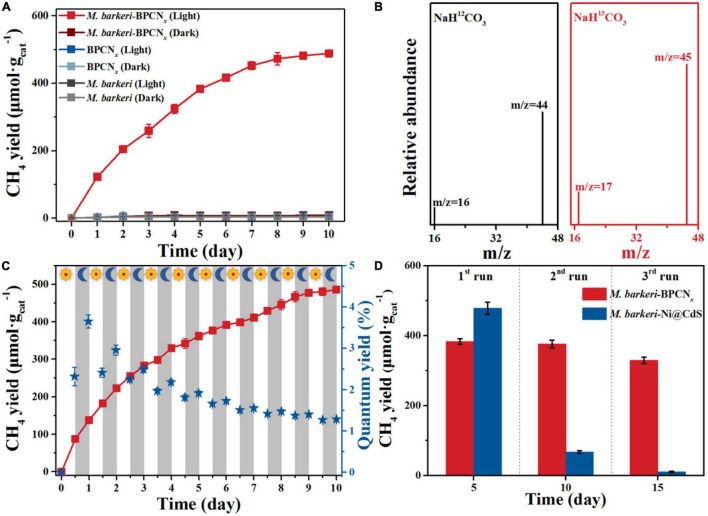
Utility of *M. barkeri*-BPCN*_*x*_* in the photoreduction of CO_2_ to CH_4_. **(A)** CH_4_ yield of *M. barkeri*-BPCN*_*x*_* and control groups. **(B)** C-labeled plot. **(C)** CH_4_ yield of *M. barkeri*-BPCN*_*x*_* with light:dark cycles of 12 h. **(D)** Multi-cycle CH_4_ yield of *M. barkeri*-Ni@CdS and *M. barkeri*-BPCN*_*x*_*.

The methanogenic performance of *M. barkeri*-BPCN*_*x*_* was measured to further explore the transmission and utilization of photogenerated electrons in the system. The key driving factors for the photocatalytic production of CH_4_ in *M. barkeri*-BPCN*_*x*_* were studied by designing related control experiments. As shown in Figure 4A, *M. barkeri* produced trace amounts of CH_4_ (4.30 ± 0.04 μmol g_cat_^–1^) under dark and light conditions, which can be traced to the intermediate metabolites of *M. barkeri* remaining in the culture process. Although the photocatalytic properties of BPCN*_*x*_* have been widely reported (Lei et al., 2018), the BPCN*_*x*_* system in this study hardly produced CH_4_ under light or dark conditions. This was probably due to the photogenerated electrons generated by BPCN*_*x*_* excited under light irradiation were stored in the substance. The system lacked the co-catalysts or electron capture agents needed to transport and utilize photoelectrons to drive the corresponding redox reactions (Lau et al., 2017; Liu et al., 2018). Interestingly, the BPCN*_*x*_* system with *M. barkeri* added under light irradiation produced CH_4_. With the extension of the irradiation time, the CH_4_ yield of the *M. barkeri*-BPCN*_*x*_* gradually increased, reaching 472.21 ± 18.87 μmol g_cat_^–1^ after 8 days. Due to the gradual oxidation of cysteine as a sacrificial reagent in the system, resulting in a lack of additional sacrificial reagents as electron donors, the CH_4_ yield of *M. barkeri*-BPCN*_*x*_* reached a maximum after 10 days of light irradiation (Yang et al., 2019). Under dark conditions, the CH_4_ yield of *M. barkeri*-BPCN*_*x*_* hardly changed, which further clarified why BPCN*_*x*_* could not produce CH_4_ under light conditions and also revealed that the CH_4_ production process with *M. barkeri*-BPCN*_*x*_* required light. To further confirm the source of CH_4_, ^13^C-labeled NaHCO_3_ was used as the carbon source and electron acceptor to carry out isotopic labeling experiments. It was found that only the characteristic peaks of ^13^CH_4_ (m/z = 17) and ^13^CO_2_ (m/z = 45) were detected (Figure 4B), indicating that CH_4_ produced by the *M. barkeri*-BPCN*_*x*_* came from CO_2_ reduction.

To simulate the day:night cycle, a light:dark alternate cycle (12 h:12 h) experiment was used to study the methanogenesis performance of *M. barkeri*-BPCN*_*x*_*. As shown in Figure 4C, the CH_4_ yield of *M. barkeri*-BPCN*_*x*_* gradually increased after each light:dark cycle, stabilizing at 485.98 ± 6.36 μmol g_cat_^–1^ after 10 cycles. It was worth noting that the peak quantum yield reached 3.65 ± 0.16%, significantly higher than that of other previously reported BAHs, which ranged from 0.59 to 2.86% (Ran et al., 2018; Fang et al., 2020). Interestingly, the CH_4_ yield in the system showed an increasing trend during both light and dark periods, and in some dark periods, the CH_4_ yield was greater than or equal to that of the light period. This might be due to how the photoelectrons produced by *M. barkeri*-BPCN*_*x*_* were stored in the system under light conditions, with the slow release of photoelectrons under dark conditions continuously driving the reaction system to reduce CO_2_ to CH_4_. To explore the multi-cycle methanogenesis performance of the BAHs constructed from metals or non-metals, *M. barkeri*-Ni@CdS with the highest reported CH_4_ yield among BAHs was selected for comparison with the *M. barkeri*-BPCN*_*x*_* constructed in this study. As shown in Figure 4D, although the CH_4_ yield of *M. barkeri*-Ni@CdS was higher than that of *M. barkeri*-BPCN*_*x*_* in the first cycle of the reaction, the CH_4_ yield of *M. barkeri*-BPCN*_*x*_* exceeded that of *M. barkeri*-Ni@CdS from the second cycle onward. On the 10th day of the reaction, the CH_4_ yield of *M. barkeri*-BPCN*_*x*_* reached 375.54 ± 11.34 μmol g_cat_^–1^, which was significantly higher than that of *M. barkeri*-Ni@CdS (67.47 ± 3.92 μmol g_cat_^–1^). As the reaction cycle progressed, the CH_4_ yield of *M. barkeri*-Ni@CdS remained basically unchanged. Notably, the CH_4_ yield of *M. barkeri*-BPCN*_*x*_* reached 1087.45 ± 29.14 μmol g_cat_^–1^ after three cycles, 1.96 times higher than that of *M. barkeri*-Ni@CdS. The results showed that *M. barkeri*-BPCN*_*x*_* had excellent methanogenesis performance and could achieve the sustainable reduction of CO_2_ to CH_4_ when the system contained sufficient sacrificial reagents.

### Maintaining the Stability of *M. barkeri*-BPCN*_*x*_*

**FIGURE 5:**
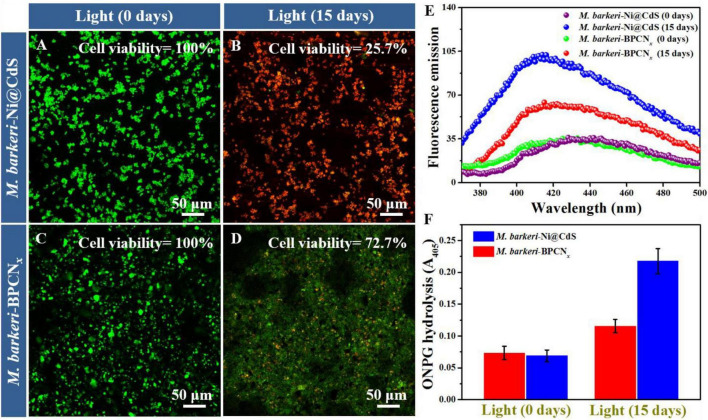
Characterizedactivity of *M. barkeri*-Ni@CdS and *M. barkeri*-BPCN*_*x*_*. **(A–D)** CLSM images of *M. barkeri*-Ni@CdS and *M. barkeri*-BPCN*_*x*_*. **(E)** Fluorescence intensity of the NPN of *M. barkeri*-Ni@CdS and *M. barkeri*-BPCN*_*x*_*. **(F)** ONPG hydrolysis absorbance of *M. barkeri*-Ni@CdS and *M. barkeri*-BPCN*_*x*_*.

To explore the reasons for the circulation, *M. barkeri* activity and the cell permeability of *M. barkeri*-BPCN*_*x*_* and *M. barkeri*-Ni@CdS in different reaction cycles were measured (Figure 5). First, the cell viability was characterized by live/dead fluorescent staining and TTC methods (Supplementary Figure 5). As shown in Figures 5A,C, in the initial stage of the reaction (Day 0), the CLSM images of *M. barkeri*-Ni@CdS and the *M. barkeri*-BPCN*_*x*_* were both green, indicating that the *M. barkeri* in both systems were living cells with the same activity (Cell activity = 100%). However, after 15 days of light reaction, the CLSM images of the *M. barkeri*-Ni@CdS (Figure 5B) changed from green to red, indicating that the *M. barkeri* in the system were cells that were nearly dead with no methanogenic activity (Cell viability = 25.7%). These results were consistent with the periodic CH_4_ production data. It is likely that the metal photocatalysts, being prone to photocorrosion, released heavy metals and poisoned the cells during the long-term photoreaction (Sakimoto et al., 2018; Ye et al., 2021). Moreover, metals such as Cd can inhibit the electron transport chain and induce the production of reactive oxygen species (ROS), thereby causing oxidative damage to the cells (Wang et al., 2004). Unexpectedly, the CLSM image of *M. barkeri*-BPCN*_*x*_* (Figure 5D) appeared green in general, indicating that *M. barkeri* was still active in the system (Cell activity = 72.7%). This can be attributed to the non-metallic elements contained in BPCN*_*x*_*, which protect *M. barkeri* while overcoming the defects of the metal photocatalyst (Xie et al., 2022). Selective cell permeability is an important function in microbial cells that is used to perform functional metabolism and can slow the entry of harmful substances into cells while allowing nutrients to enter the cells (Chen, 2007). Thus, cell permeability is an important indicator for the characterization of cell viability. In this study, the cell permeability of *M. barkeri*-BPCN*_*x*_* and *M. barkeri*-Ni@CdS were measured by NPG hydrolysis and NPN uptake experiments under light excitation. As shown in Figures 5E,F, compared with *M. barkeri*-BPCN*_*x*_*, *M. barkeri*-Ni@CdS showed a stronger fluorescence signal and absorbance after 20 days of light reaction. In a related study, the metal nano-zinc oxide generated ROS to destroy the cell membrane structure of *Escherichia coli*, inhibiting the protein activity at the membrane and eventually leading to the death of the cell (Padmavathy and Vijayaraghavan, 2011). The results showed that *M. barkeri*-BPCN*_*x*_* had lower cell permeability under light conditions, which was beneficial to maintaining cell function and metabolic activity. This may be due to BP nanosheets acting as antioxidants to reduce the toxic ROS formation outside the cells, thereby decreasing harmful substances from entering the cells (Das et al., 2017; Chen et al., 2018). The above results revealed a possible reason for the multi-cycle methanogenesis properties of *M. barkeri*-BPCN*_*x*_*: The system had maintained cell permeability under light irradiation, effectively reducing the damage to *M. barkeri* while helping to preserve the long-term activity of *M. barkeri*. The stability of *M. barkeri*-BPCN*_*x*_* could then be maintained to drive the reduction of CO_2_ to CH_4_.

## Conclusion

In this study, metal-free photocatalysts (BPCN*_*x*_*) were combined with *M. barkeri* for the successful construction of *M. barkeri*-BPCN*_*x*_*, and the methanogenesis performance was evaluated. Under visible light, the introduction of BP facilitated the separation of CN*_*x*_* photogenerated charges and enhances the quantum yield, providing a sustained energy source for cyclically driven *M. barkeri* to reduce CO_2_ to CH_4_. Impressively, the BPCN*_*x*_* maintained high cellular activity and achieved a total CH_4_ yield of 1087.45 ± 29.14 μmol g_cat_^–1^ after three cycles, 1.96 times higher than that of the *M. barkeri*-Ni@CdS systems. The cyclic stability was likely achieved through overcoming the defects of the metal photocatalyst and the retention of cell permeability, thereby effectively maintaining the activity of *M. barkeri*. These results highlight the core role of the metal-free BPCN*_*x*_* photocatalysts in the construction of BAHs and are of great significance for the development of environmentally-friendly, low-cost, and efficient BAHs.

## Data Availability Statement

The original contributions presented in this study are included in the article/Supplementary Material, further inquiries can be directed to the corresponding authors.

## Author Contributions

AH provided concept, performed experiment, conducted the data analyses, and wrote the original draft. TF and GR conducted the data analyses and reviewed this manuscript. MZ and WY assisted in methodology designing and performed Experiment. SNZ conducted the data analyses and reviewed this manuscript. SGZ reviewed this manuscript and provided funding acquisition. All authors contributed to the article and approved the submitted version.

## Conflict of Interest

The authors declare that the research was conducted in the absence of any commercial or financial relationships that could be construed as a potential conflict of interest.

## Publisher’s Note

All claims expressed in this article are solely those of the authors and do not necessarily represent those of their affiliated organizations, or those of the publisher, the editors and the reviewers. Any product that may be evaluated in this article, or claim that may be made by its manufacturer, is not guaranteed or endorsed by the publisher.
