# Supplementary material for: Sustained Biotic-Abiotic Hybrids Methanogenesis Enabled Using Metal-Free Black Phosphorus/Carbon Nitride
Source: Front Microbiol. 2022 Jul 12;13:957066. doi: 10.3389/fmicb.2022.957066 (PMC9314768; doi:10.3389/fmicb.2022.957066)
Supplement: Supplementary file 1 [file Data_Sheet_1.pdf]

Supplementary Material

Andong Hu [1], Tao Fu[1], Guoping Ren[1], Minghan Zhuang[1], Weiqi Yuan[1], Sining Zhong[1] and Shungui Zhou[1**]*

[1] *Fujian Provincial Key Laboratory of Soil Environmental Health and Regulation, College of Resources and Environment, Fujian Agriculture and Forestry University, Fuzhou 350002, China*

1.1 Supplementary Figures

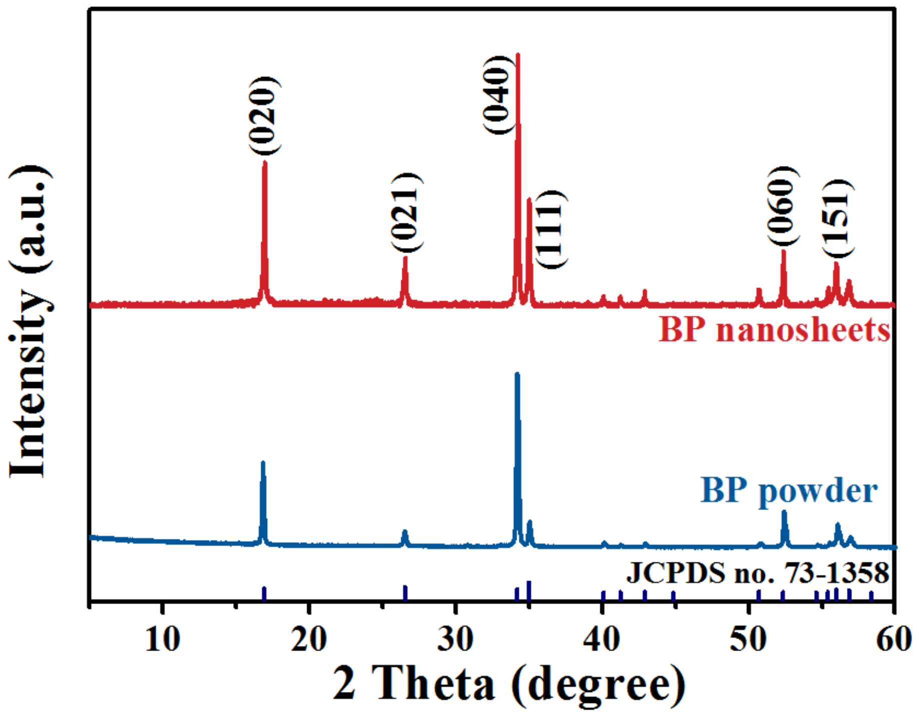

FIGURE S1 | The XRD patterns of BP powder and nanosheets.

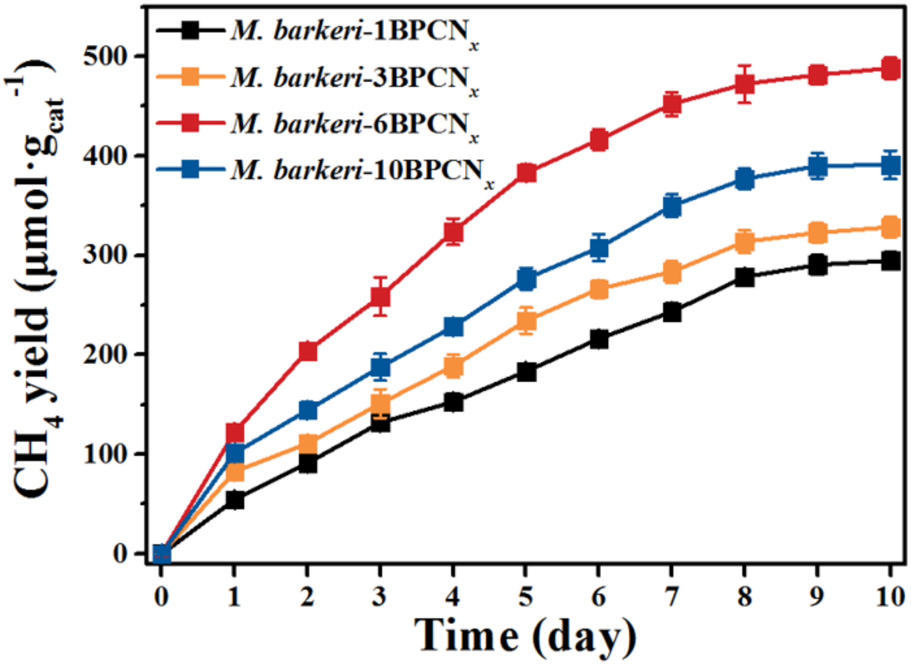

FIGURE S2 | CH_4 yields for different weight ratios of BP to CN_x (1, 3, 6, and 10 wt% expressed as $1BPCN_x$, $3BPCN_x$, $6BPCN_x$, and $10BPCN_x$, respectively).

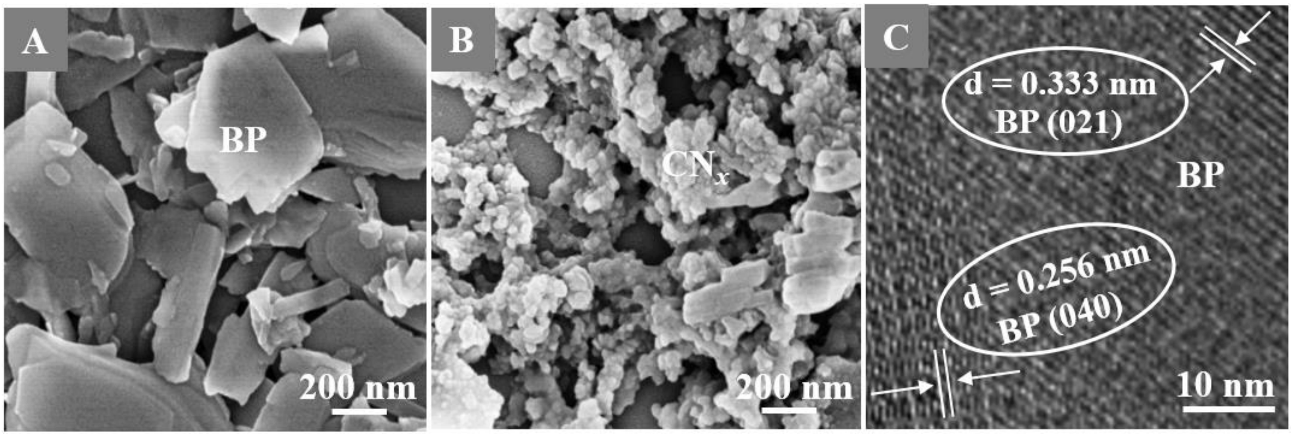

FIGURE S3 | (A, B) SEM images of BP and CN$_x$; **(C)** TEM images of BP.

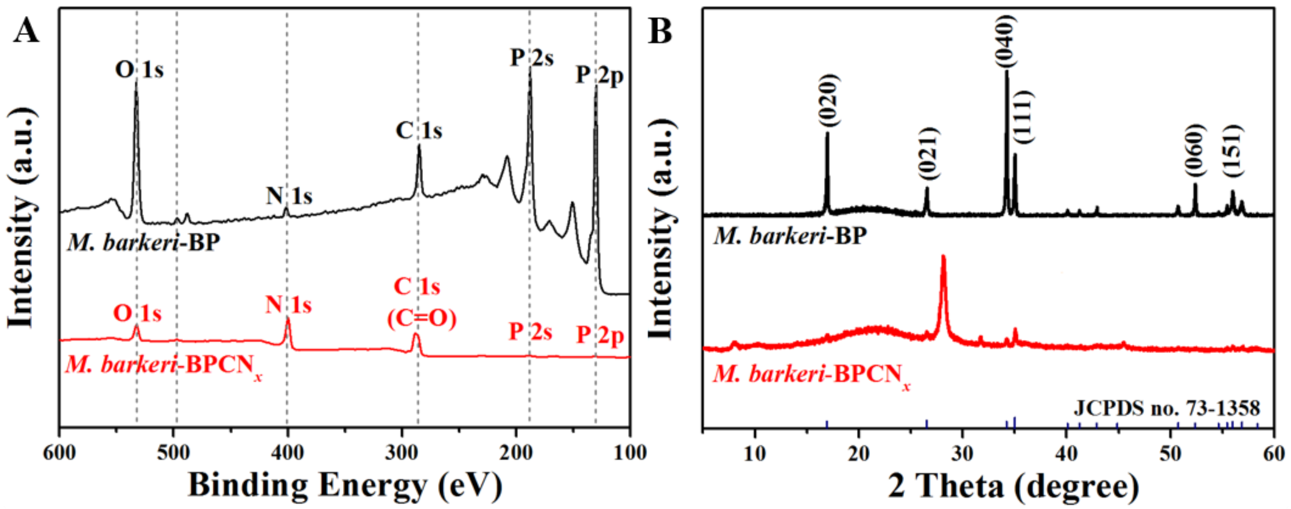

FIGURE S4 | (A) High-resolution XPS spectra; **(B)** XRD patterns.

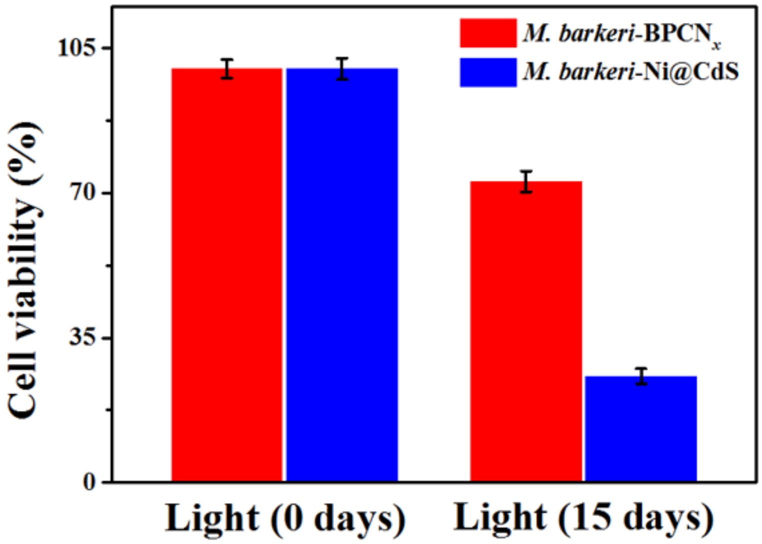

FIGURE S5 | (A) Cell viability of *M. barkeri*-Ni@CdS and *M. barkeri*-BPCN$_x$.

1.2 Supplementary Tables

TABLE S1 | Composition of sterilized heterotrophic medium (SHM) and sterilized autotrophic medium (SAM).

Component	SHM (g/L)	SAM (g/L)
$MgCl_2 \cdot 6H_2O$	0.4	0.4
$CaCl_2 \cdot 2H_2O$	0.1	0.1
NH_4Cl	0.1	0.1
KH_2PO_4	0.2	0.2
KCl	0.5	0.5
HEPES	7.16	7.16
$NaHCO_3$	2.52	2.52
$Na_2S \cdot 9H_2O$	0.24	-
NaAc	1.394	-
Cysteine-HCl	-	0.24
Trace element solution SL-10*	1 mL	1 mL
Selenite-tungastate solution**	1 mL	1 mL
Vitamin solution***	3 mL	3 mL

*Per liter, the medium containing

HCl (2M)	50 mL
$FeCl_2 \cdot 4H_2O$	2 g
$ZnCl_2$	0.2 g

$MnCl_2 \cdot 4H_2O$	0.1 g
H_3BO_3	0.18 g
$CoCl_2 \cdot 6H_2O$	0.05 g
$CuCl_2 \cdot 2H_2O$	6 mg
$NiCl_2 \cdot 6H_2O$	72 mg
$Na_2MoO_4 \cdot 2H_2O$	108 mg

**Per liter, the medium containing

NaOH	0.5 g
$Na_2SeO_3 \cdot 5H_2O$	3 mg
$Na_2WO_4 \cdot 2H_2O$	4 mg

***Per liter, the medium containing

4-aminobenzoic acid	0.04 g
D(+)-biotin	0.01 g
DL-a-lipoic acid	0.01 g
Calcium-D(+)-panto-thenate	0.1 g
Pyridoxine-HCl	0.1 g
Folic acid	0.03 g
Nicotinic acid	0.05 g
Riboflavin	0.05 g

Thiamin-HCl·$2H_2O$	0.01 g
Vitamin B_{12}	0.05 g
